# The vesicle transport gene SEC23A is a novel prognostic indicator and therapeutic target in gastric cancer

**DOI:** 10.3389/fonc.2026.1728472

**Published:** 2026-04-13

**Authors:** Liang Li, Guanglong Chen, Weijie Zhao, Zikun Wu, Chai Lv, Ye Kong, Huihan Ai, Hang Yang, Zhi Li

**Affiliations:** The Affiliated Cancer Hospital of Zhengzhou University & Henan Cancer Hospital, Zhengzhou, China

**Keywords:** biomarker, gastric cancer, proliferation, SEC23A, therapeutic target, tumor immune microenvironment

## Abstract

**Objective:**

SEC23A, a gene implicated in vesicle transport, has an undefined role in the progression of gastric cancer (GC). This study aims to comprehensively characterize the clinical significance, molecular mechanisms, and therapeutic potential of SEC23A in GC.

**Methods:**

We systematically analyzed SEC23A expression and its prognostic value in GC cohorts from The Cancer Genome Atlas and Gene Expression Profiling Interactive Analysis. Genetic alterations were assessed using cBioPortal. Functional networks, including competitive endogenous RNA and protein-protein interaction networks, were constructed. The tumor immune microenvironment was evaluated using the Tumor Immune Estimation Resource 2.0, CIBERSORT, and single-sample gene set enrichment analysis. Drug sensitivity was predicted using data from The Cancer Immunome Atlas and the R package “pRRophetic”. Key findings were validated with data from the Gene Expression Omnibus and the Human Protein Atlas. *In vitro* functional assays were conducted to assess the impact of SEC23A knockdown on GC cell proliferation.

**Results:**

Overexpression of SEC23A was significantly associated with poor overall survival in GC patients, particularly in those with undifferentiated tumors. Functionally, high SEC23A levels promoted tumor cell proliferation and were correlated with an immunosuppressive microenvironment, characterized by increased M2 macrophage infiltration and reduced Tregs. Patients with high SEC23A expression exhibited reduced sensitivity to anti-PD-1/CTLA-4 immunotherapy but showed heightened sensitivity to certain small-molecule inhibitors. Consistent with these observations, *in vitro* experiments confirmed that SEC23A knockdown effectively suppressed GC cell proliferation.

**Conclusions:**

Our findings underscore the critical role of SEC23A in driving GC progression and modulating the immune landscape. SEC23A represents a promising prognostic biomarker and a novel therapeutic target for GC, potentially guiding strategies involving immunotherapy and small-molecule inhibitors.

## 1 Introduction

Gastric cancer (GC), the most common malignancy worldwide, was responsible for over one million new cases and 760,000 deaths in 2020, equating to approximately one in thirteen fatalities globally (1). Furthermore, it exhibits the fourth-highest incidence rate globally and ranks second in terms of mortality rate (1, 2). Despite substantial progress in research on gastric cancer in recent years, patients still face an unfavorable prognosis. As such, it is imperative to deepen our understanding of the underlying mechanisms that drive disease progression and to identify new prognostic factors and therapeutic strategies. These efforts are crucial for improving long-term survival outcomes for patients battling this disease.

The protein encoded by SEC23A is a cytoplasmic protein that plays a crucial role in mediating vesicle transport, particularly in facilitating the transport process between the endoplasmic reticulum and the Golgi apparatus. Moreover, it is implicated in cellular transport and secretion (3–5). SEC23A also plays a role in regulating T cell function (6). Research has shown that SEC23A is intricately linked to cellular autophagy, a physiological process believed to be involved in the development and progression of cancer (7, 8). SEC23A is observed to be overexpressed in various aggressive tumor types, namely, prostate cancer (9), colorectal cancer (10), melanoma (11), and breast cancer (12). These observations suggest that SEC23A may represent a promising prognostic marker and therapeutic target for a range of aggressive cancers, including gastric cancer (GC). However, the current body of research offers only limited evidence regarding the correlation between SEC23A and GC, and the precise role played by SEC23A in GC is not yet fully understood. Additionally, a deeper understanding of the regulatory pathways through which SEC23A impacts the progression of GC is still needed. Further research is required to fully elucidate the significance of SEC23A in the context of GC.

In the present research, we utilized The Cancer Genome Atlas (TCGA) database to analyze SEC23A expression in gastric cancer. The study focused on its association with clinicopathological characteristics and prognosis. Gene Set Enrichment Analysis (GSEA) was employed to investigate the potential molecular roles of SEC23A. In previous studies, few researchers have conducted studies on SEC23A-related pathways. In this study, we have determined by GSEA analysis that high SEC23A expression is associated with ‘TGF_BETA_SIGNALING_PATHWAY’, ‘MAPK_SIGNALING_PATHWAY’, and ‘CCR5_PATHWAY’. This suggests a link between dysregulated SEC23A pathways and the development and prognosis of gastric cancer. Furthermore, the investigation of SEC23A and its potential association with tumor-infiltrating immune cells was pursued in this study. The balance of immune effector cells within the tumor microenvironment is crucial for determining the evasion of tumor cells from immune responses. The infiltration of cells such as macrophages and regulatory T cells is correlated with an unfavorable prognosis. Additionally, we constructed competing endogenous RNA (ceRNA) and protein-protein interaction (PPI) networks to identify interactions between miRNA and proteins with SEC23A. Moreover, bioinformatics prediction was employed to identify potentially more effective clinical drugs for patients with high SEC23A expression.

In summary, this study sheds light on the regulatory role of non-coding RNA and potential molecular mechanisms involving SEC23A. It establishes a correlation between SEC23A and prognosis as well as immune infiltration in gastric cancer. Furthermore, it identifies potentially more effective drugs for patients with high SEC23A expression and enhances our understanding of possible pathways involved in gastric carcinogenesis.

## 2 Materials and methods

### 2.1 Biospecimen collection

In total, nine fresh gastric cancer tissue samples were acquired from the Affiliated Cancer Hospital of Zhengzhou University & Henan Cancer Hospital from December 2021 to December 2022. These specimens were confirmed to be gastric adenocarcinomas through postoperative pathology. The T stage of the samples was distributed as follows: three cases in the T2 stage, three cases in the T3 stage, and three cases in the T4 stage. Written informed consent was obtained from the patients, and approval was granted by the internal review and ethics boards (Approval No. 2021-499-002). The inclusion criteria were as follows: (1) clinical diagnosis of gastric cancer; (2) complete clinical data and postoperative pathological report for each patient; and (3) informed consent from the patient.

### 2.2 Cell culture

The undifferentiated gastric cancer cell line HGC-27 (RRID: CVCL_1279), the poorly differentiated gastric cancer cell lines MKN-45 (RRID: CVCL_0434) and NCI-N87 (RRID: CVCL_1603), and AGS (RRID: CVCL_0139) were acquired from the Institute of Medical Biotechnology, Chinese Academy of Medical Sciences & Peking Union Medical College. Cell lines underwent STR profiling to verify their authenticity. The cells were cultured in RPMI1640 medium (Thermo Fisher Scientific, USA) supplemented with 10% fetal bovine serum (Thermo Fisher Scientific) at 37 °C in a 5% CO_2_ atmosphere within a CO_2_ incubator.

### 2.3 SEC23A mRNA expression analysis

Gene Expression Profiling Interactive Analysis (RRID: SCR_018294) provides useful functions, such as differential expression analysis, survival analysis, and identification of similar genes. Consequently, it serves as an efficient tool for comprehensively exploring the TCGA and GTEx datasets (13). The GEPIA2 database was utilized to evaluate the expression of SEC23A in diverse cancer types, and the plots were generated using the browser. Furthermore, we accessed two distinct datasets, denoted as GSE54129 and GSE118916, from the Gene Expression Omnibus (GEO) (RRID: SCR_005012) database, located at https://www.ncbi.nlm.nih.gov/geo. These datasets encompassed valuable samples from both gastric cancer (GC) tissue and normal gastric tissue. By utilizing the R software (version 4.2.1), we conducted an analysis examining the differentially expressed levels of SEC23A in GC tissue and normal gastric tissue. The findings were then visualized, yielding results that shed light on our investigation.

### 2.4 Overall survival analysis

The TCGA and GEO databases were analyzed to generate survival curves and evaluate the expression of SEC23A across diverse cancer tissues, while also assessing its prognostic significance. Notably, the Kaplan-Meier plotter online database (http://kmplot.com/analysis/) was employed to discern the impact of individual genes on the survival of a substantial cohort of gastric cancer patients (n = 881).

### 2.5 Independent prognostic factors analysis

Survival data for 450 patients with GC were obtained from UCSC Xena (https://xena.ucsc.edu/). Exclusion criteria included: (1) missing critical data and (2) non-cancerous data. Statistical analyses were performed using R software (version 4.2.1). Single-factor and multi-factor Cox regression analyses were conducted to identify independent prognostic factors, contributing to a comprehensive understanding of GC progression and disease outcome.

### 2.6 Identification of differential genes and GO enrichment analysis

The data used in this study were downloaded from the TCGA database via UCSC Xena. To characterize the impact of SEC23A mRNA expression, the data were divided into two groups based on the median mRNA level of SEC23A: high expression and low expression groups. Subsequently, differential gene analysis was conducted to identify genes that exhibited significant differences between these two groups. The analysis utilized the “limma” package, and specific criteria were defined, including a False Discovery Rate (FDR) threshold of less than 0.05 and an absolute log2 fold change (|log2FC|) greater than or equal to 2. To visually represent the results, a volcano plot was generated using the “ggplot2” package. Furthermore, Gene Ontology (GO) enrichment analysis was performed using R software (version 4.2.1) to gain insights into the biological functions and processes associated with the differentially expressed genes. The resulting enriched GO terms were then visualized to facilitate interpretation and understanding of the molecular mechanisms underlying the observed gene expression differences.

### 2.7 Gene set enrichment analysis

In the Gene Set Enrichment Analysis (GSEA), data were obtained from the TCGA database via UCSC Xena. The dataset consisted of 450 patients diagnosed with GC, and they were divided into high- and low-expression groups based on the median expression value of SEC23A. From each group, the top 10% of samples (n = 90) were selected for further analysis. GSEA (version 4.2.3) software was used to detect pathways enriched among the top-ranked genes in both groups. For each analysis, the number of gene set alignments was set to 1000. The nominal (NOM) p-value, false discovery rate (FDR), and normalized enrichment score (NES) were used to identify the enriched pathways in each phenotype.

### 2.8 Protein-protein interaction network

To construct the protein-protein interaction (PPI) network, we used the Search Tool for the Retrieval of Interacting Genes (STRING) database, version 11.5 (https://string-db.org/) (14). The STRING database is one of several online resources dedicated to organism-wide protein association networks, providing a comprehensive platform for the exploration and analysis of protein-protein interactions. The GeneMANIA database was utilized for the analysis of both physical and functional interactions.

### 2.9 Correlation analysis of SEC23A with immune infiltrating

For the analysis of the correlation between SEC23A and immune infiltration, the Timer 2.0 database was utilized (15). Single-sample gene set enrichment analysis (ssGSEA) was performed using the “GSVA” package to assess immune cell infiltration and associated immune-related pathways. This analysis helps determine the enrichment scores for immune-related gene sets or signatures in each sample, providing an estimation of the activity levels of specific immune cell types and immune-related pathways. In addition, to further clarify the subtypes of different immune cells, the relative abundance of 22 types of immune cells in the TCGA database was quantitatively analyzed using the CIBERSORT algorithm. By comparing the high- and low-SEC23A-expression groups, differences in the infiltration of these immune cell types were quantitatively analyzed.

### 2.10 Gene mutation analysis and ceRNA network construction

Gene mutation analysis of *SEC23A* was performed using the cBioPortal database. The cBioPortal is an online resource that provides comprehensive and interactive analyses of cancer genomics datasets. For the prediction of miRNAs targeting SEC23A, we employed the STARBASE database (16). The STARBASE database is an open-source platform specifically designed for studying miRNA-target interactions and RNA-RNA interactions. The selection criteria were set as follows: program number > 2, correlation coefficient > 0.2, and *P* < 1.0e-6. The ceRNA network results were visualized using Cytoscape software (version 3.9.1) (17). By integrating the results from the cBioPortal, STARBASE, and Cytoscape analyses, we gained insights into the genetic mutations in SEC23A, predicted miRNAs targeting SEC23A, and the ceRNA network involving SEC23A.

### 2.11 Correlation analysis between miRNA expression levels and prognosis

To analyze the mechanism of action of miRNA regulated by SEC23A gene expression in the occurrence and development of gastric cancer, the correlation between miRNA expression levels and prognosis was analyzed using R software (version 4.2.1).

### 2.12 Potential drug prediction

Immunotherapy sensitivity data for stomach adenocarcinoma (STAD) were downloaded from the TCIA database (https://www.tcia.at/home). The results were visualized as a violin plot using GraphPad Prism 8. The half-maximal inhibitory concentration (IC50) of the drugs in patients with STAD was estimated using the “pRRophetic” package in R software (version 4.2.1) to predict drug efficacy.

### 2.13 Pathological and subcellular analysis of SEC23A

The Human Protein Atlas (HPA) was used to obtain immunohistochemical and immunofluorescence staining images of SEC23A in gastric adenocarcinoma and normal tissues. 

### 2.14 Reverse transcription and quantitative real-time polymerase chain reaction

Total RNA was isolated using reagents from Nanjing Vazyme Biotech Co., Ltd., in accordance with the manufacturer’s instructions. PCR reactions were performed in 20 µl reaction volumes, also in accordance with the manufacturer’s instructions (Q711-02/03, Vazyme Biotech Co., Ltd.). The relative expression level of SEC23A was normalized to the level of GAPDH using the 2^-△△Ct^ method. The primer sequences used in the experiment are as follows (**Table 1**):

**Table 1:** Primer sequences

*Gene*	Forward sequence	Reverse sequence
*SEC23A*	5’- AGTGGCGGAAGTCAGGATAC -3’	5’- GGCATTGGAAATCTGGAGTG -3’
*GAPDH*	5’- CATGAGAAGTATGACAACAGCCT -3’	5’- AGTCCTTCCACGATACCAAAGT -3’

### 2.15 Plate clone formation experiment

The plate clone formation experiment is a commonly used method to assess cell proliferation capacity and clonogenic potential. In this study, the SEC23A protein was knocked down to investigate changes in the proliferation and clonogenic capabilities of gastric cancer cell lines. A total of 500 cells were seeded in 6-well culture plates and incubated for 12 h. Then, si-SEC23A (Beyotime Biotechnology, China) was used to knock down the SEC23A protein in human gastric cancer cell lines (AGS, HGC-27), and the cells were continuously cultured for two weeks. The cells were fixed with paraformaldehyde for 30 min, stained with hematoxylin for 15 min, washed with PBS, and cell clone images were captured. The number of clones was counted to calculate the clonogenic efficiency.

### 2.16 Cell proliferation experiment

The EdU cell proliferation assay is utilized to investigate the DNA synthesis activity and proliferative capacity of cells *in vitro*. EdU (5-ethynyl-2’-deoxyuridine) is an analog of thymidine, capable of being taken up by cells and incorporated into the nuclei undergoing DNA synthesis. This experiment employed the BeyoClick EdU-555 Cell Proliferation Assay Kit (C0075S, Beyotime, China). By detecting the number and localization of cells marked with EdU, the proliferative activity of cells can be assessed. A total of 50,000 cells were cultured overnight in 12-well plates, followed by siRNA-mediated knockdown of the SEC23A protein in human gastric cancer cell lines (AGS, HGC-27). The cells were then incubated at 37 °C with 5% CO_2_ for 48 h prior to conducting the EdU cell proliferation assay. The results were analyzed under a fluorescence microscope.

### 2.17 Statistical analysis

The statistical analyses were conducted using R software (version 4.2.1). The independent samples t-test was employed to analyze the differential expression levels of SEC23A mRNA between gastric cancer (GC) tissues and adjacent normal tissues. Univariate and multivariate analyses were performed using the Cox proportional hazards regression model. Statistically significant differences were considered when *P* < 0.05 (***P < 0.001, **P < 0.01, *P < 0.05).

## 3 Results

### 3.1 SEC23A expression is upregulated in GC and correlates with poor prognosis

**Figure 1:**
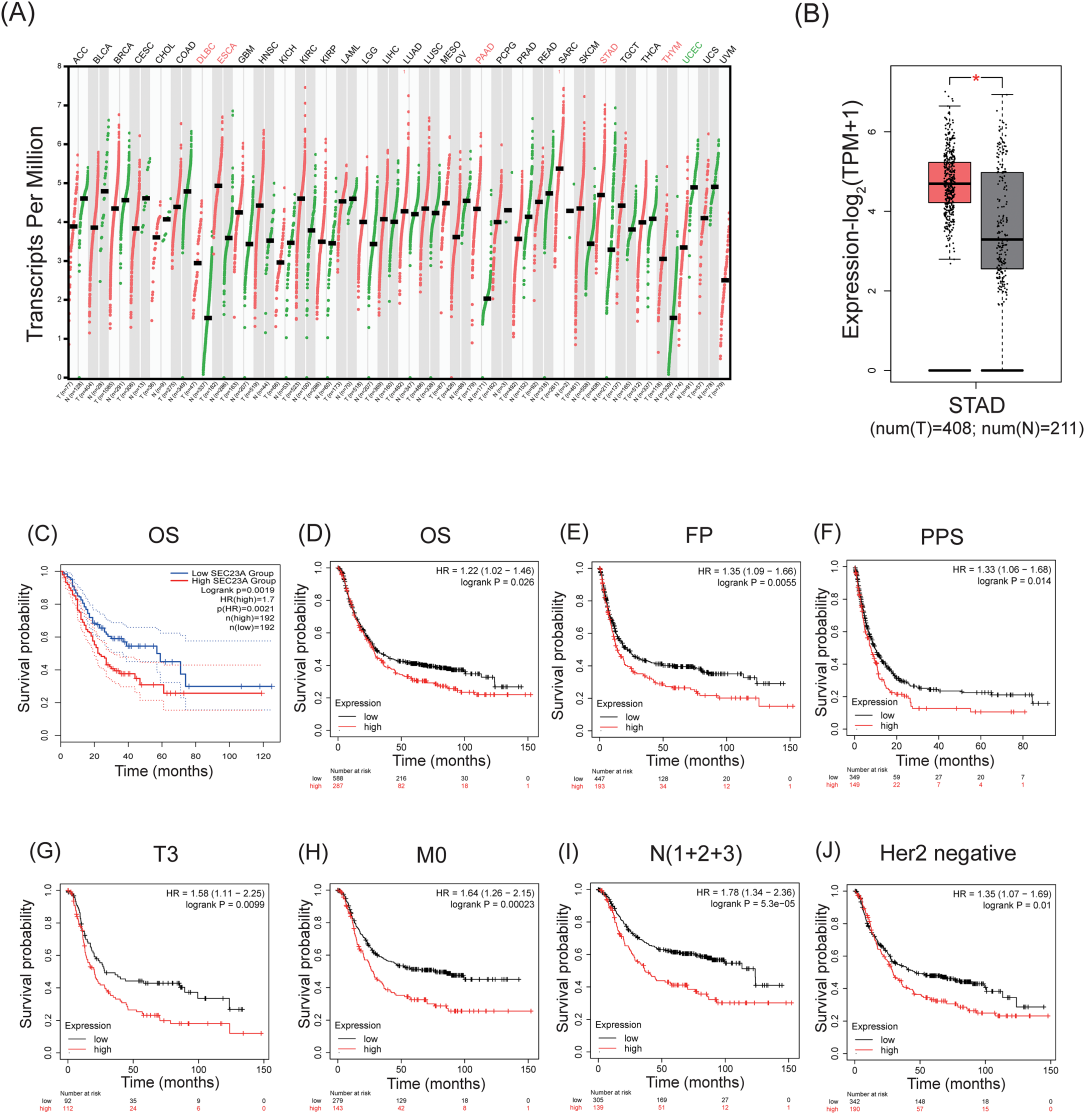
Upregulation of SEC23A expression in GC and correlation with poor prognosis. **(A)** SEC23A expression in different tumor types from TCGA data analyzed in GEPIA2. **(B)** High expression of SEC23A in STAD. **(C)** Survival analysis of SEC23A using GEPIA2. **(D-J)** Survival analysis of SEC23A using the Kaplan-Meier plotter database.

The study utilized the GEPIA2 database to assess the transcript levels of SEC23A across various common cancers. The analysis revealed that SEC23A mRNA expression was significantly higher in DLBC, ESCA, PAAD, STAD, and THYM, but notably reduced in UCEC (**Figures 1A, B**). Specifically focusing on gastric cancer (GC), SEC23A was found to be expressed at higher levels compared to normal tissue, indicating its potential involvement in the development and progression of GC. Additionally, the prognostic value of SEC23A was investigated using the GEPIA2 and Kaplan-Meier Plotter databases. High SEC23A expression was found to be significantly correlated with shorter overall survival (HR = 1.70, *P* = 0.0021) (**Figure 1C**) in the GEPIA2 database. Consistently, in the Kaplan-Meier database, high SEC23A expression was negatively correlated with overall survival (OS), first progression (FP), and post-progression survival (PPS) (OS, *P* = 0.026; FP, *P* = 0.0055; PPS, *P* = 0.014), especially in patients with T3, HER-2-negative, N (1 + 2 + 3) (**Figures 1D–J**). These findings suggest that SEC23A could serve as a potential prognostic biomarker for GC and may aid in personalizing treatment approaches to improve patient outcomes and quality of life.

### 3.2 SEC23A is a high-risk and independent prognostic factor in GC

**Figure 2:**
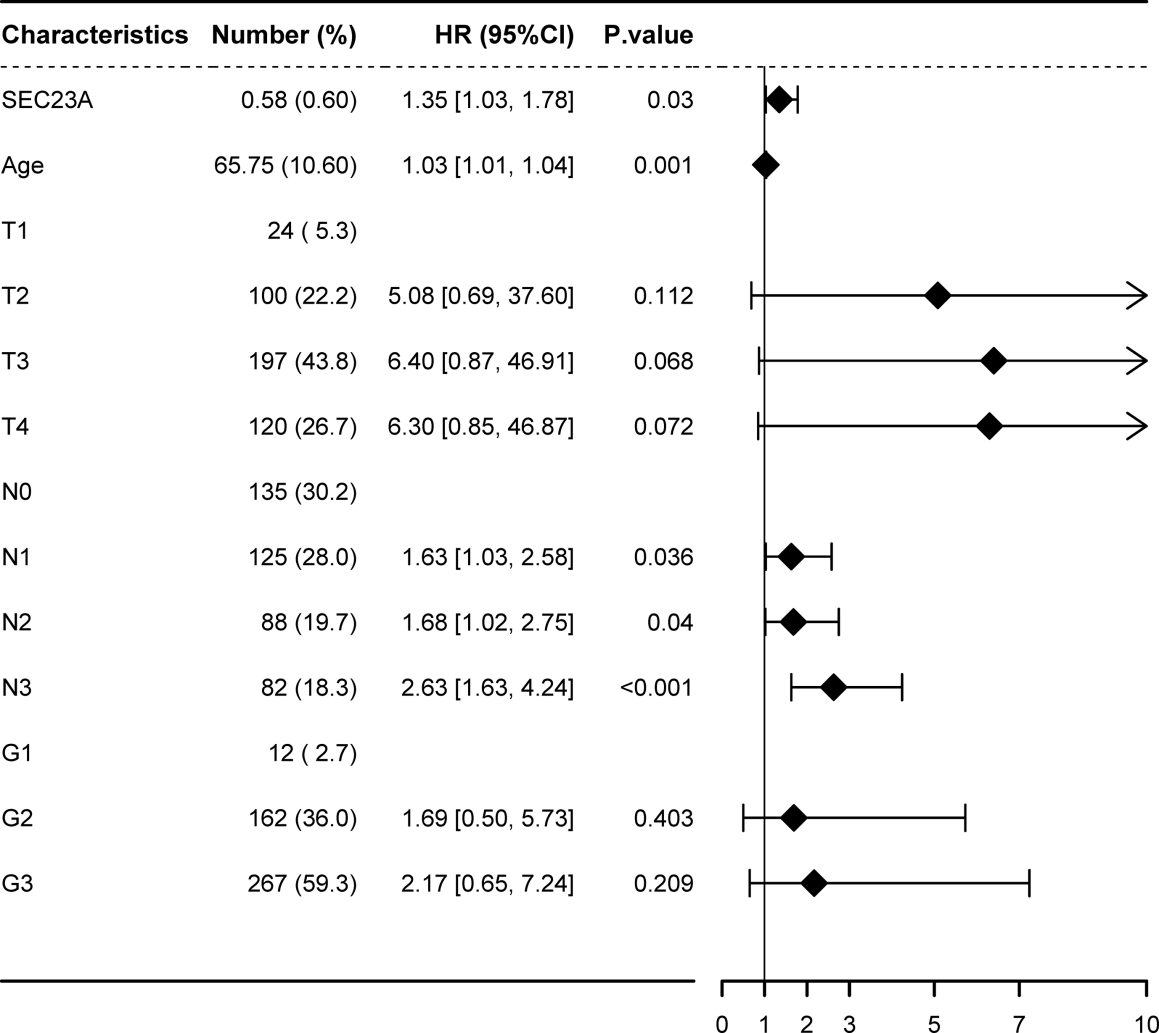
Multivariate cox analysis showing hazard ratios (HRs) of different factors.

The study conducted multivariate Cox survival analysis using clinical and gene expression data from 450 GC cases obtained from the TCGA database. The results revealed that age, N stage, and SEC23A expression were independent predictors of unfavorable prognosis in GC patients (all *P* < 0.05) (**Figure 2**). Specifically, higher SEC23A expression was found to be significantly associated with a 35% increase in overall mortality rates compared to lower expression levels. This indicates that higher SEC23A expression is linked to poorer prognosis in GC patients. In conclusion, these findings suggest that SEC23A may be an unfavorable prognostic factor and an independent prognostic marker for gastric cancer.

### 3.3 GSEA and gene ontology enrichment analysis

**Figure 3:**
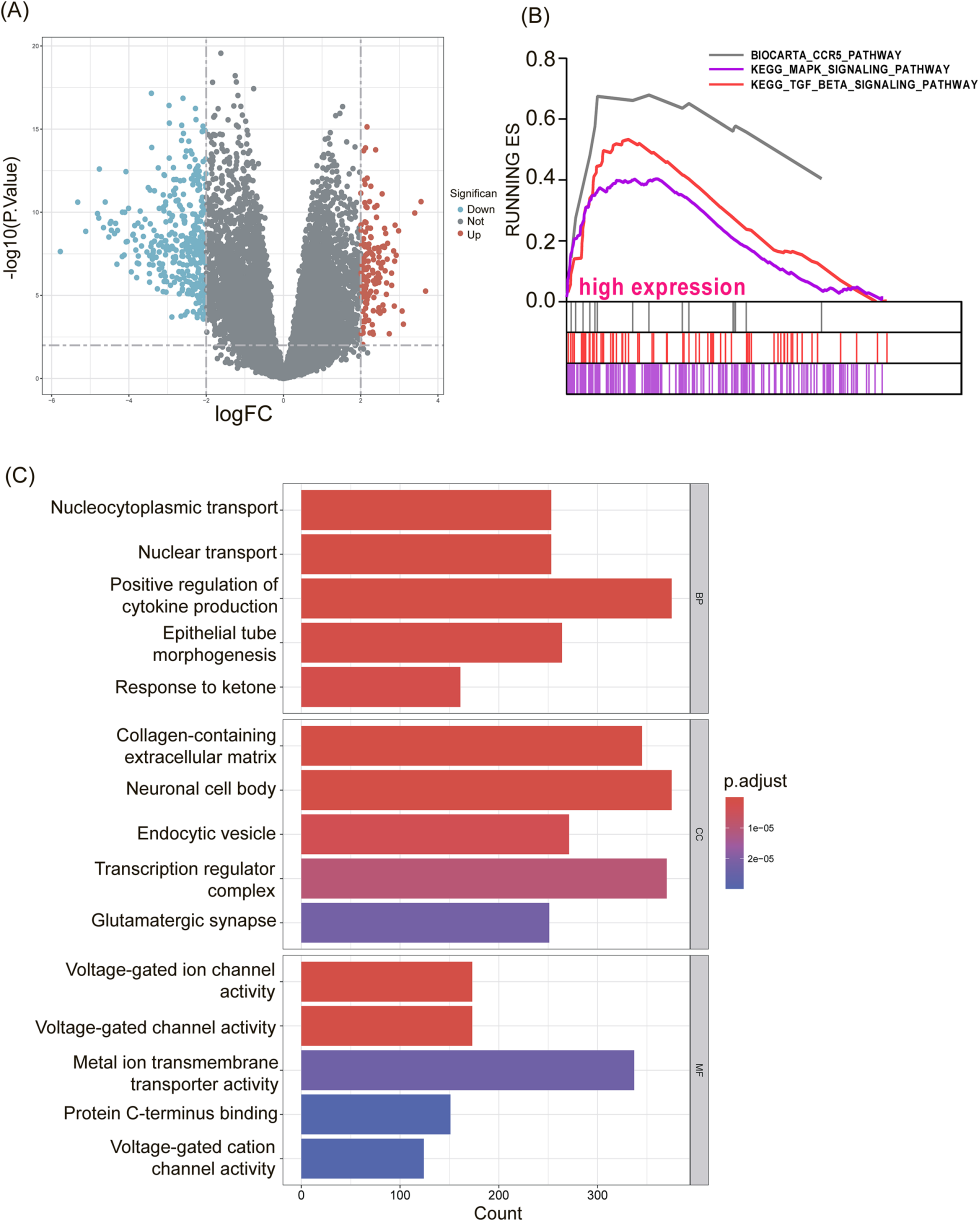
GSEA and GO enrichment analysis. **(A)** Identification of DEGs between high and low SEC23A expression groups. **(B)** Enriched pathways identified by GSEA in the high-SEC23A group. **(C)** GO enrichment analysis.

To gain further insight into the potential molecular function of SEC23A in gastric cancer (GC), the patients in the TCGA dataset were divided into two groups based on their median SEC23A mRNA levels. Differentially expressed genes (DEGs) analysis was then performed using the R package, which identified 154 positively and 367 negatively correlated genes between the SEC23A-high and SEC23A-low subgroups (**Figure 3A**). Additionally, GSEA analysis was used to predict SEC23A-related signaling pathways. Three pathways were selected from the GSEA results that were upregulated with *P* < 0.05 and FDR < 0.25, namely, ‘BIOCARTA_CCR5_PATHWAY’, ‘KEGG_TGF_BETA_SIGNALING_PATHWAY’, and ‘KEGG_MAPK_SIGNALING_PATHWAY’ (**Figure 3B**). These pathways are known to be involved in regulating cell proliferation and immunity in GC. Moreover, GO enrichment analysis was performed between samples with low and high levels of SEC23A mRNA, which suggested that the DEGs are mainly related to “voltage-gated channel activity”, “transcription regulator complex”, “positive regulation of cytokine production”, “nuclear and nucleocytoplasmic transport”, and “epithelial tube morphogenesis” (**Figure 3C**).

### 3.4 Cell experiments *in vitro*

**Figure 4:**
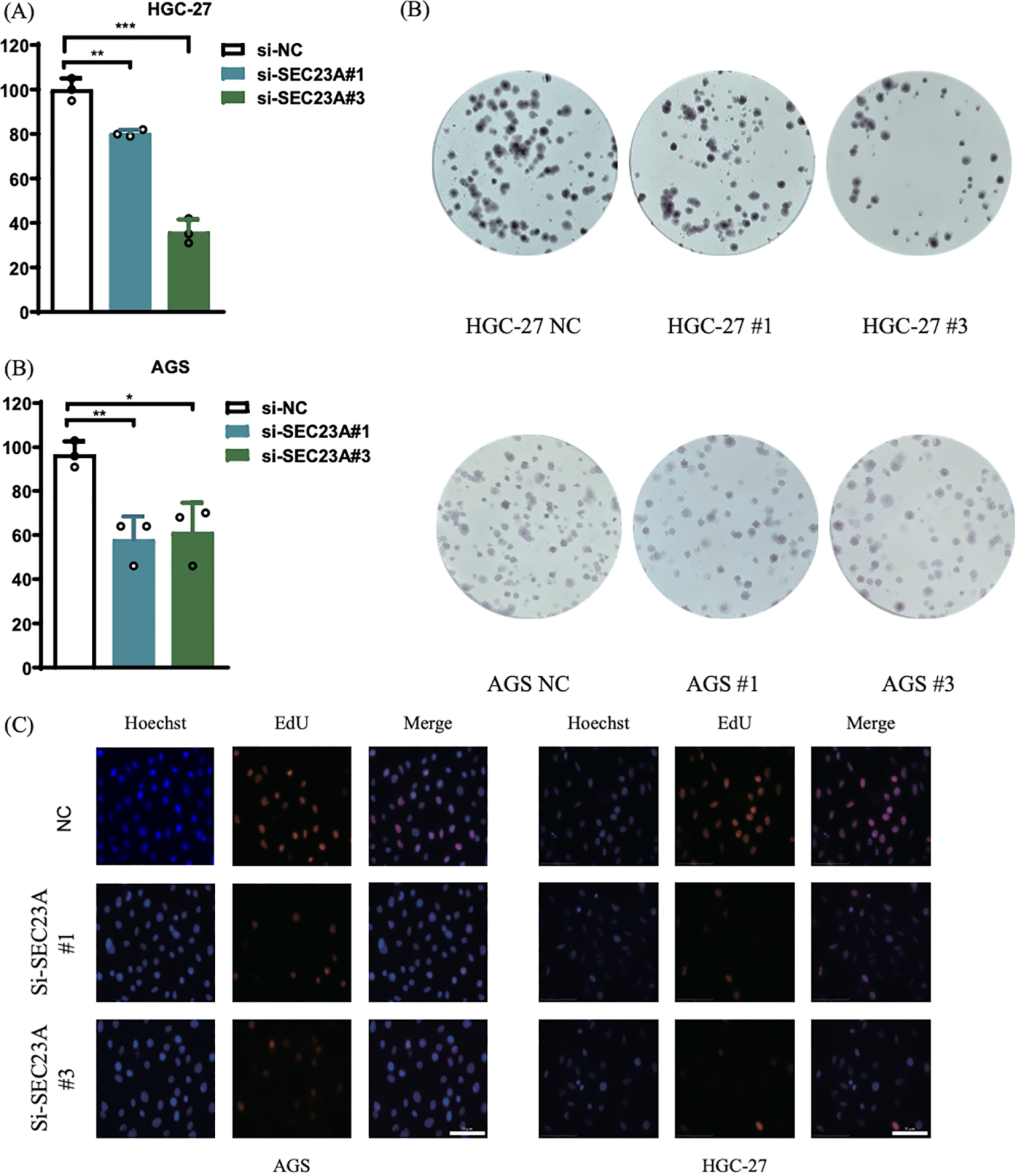
Cell experiments *in vitro*. **(A, B)** Colony formation assay. **(C)** EdU proliferation assay. ***P < 0.001, **P < 0.01, *P < 0.05.

The plate clone formation experiment evaluated the impact of SEC23A on extracellular cell growth. The results demonstrated a significant decrease in clone numbers in AGS and HGC-27 cells *in vitro* upon SEC23A knockdown (**Figures 4A, B**). Subsequently, through the use of EdU labeling for proliferating cells, quantitative analysis indicated a notable reduction in the percentage of EdU-positive cells in AGS and HGC-27 following SEC23A knockdown (**Figure 4C**). These findings suggest that inhibition of SEC23A suppresses the growth of gastric cancer cells *in vitro*.

### 3.5 Immunological significance

**Figure 5:**
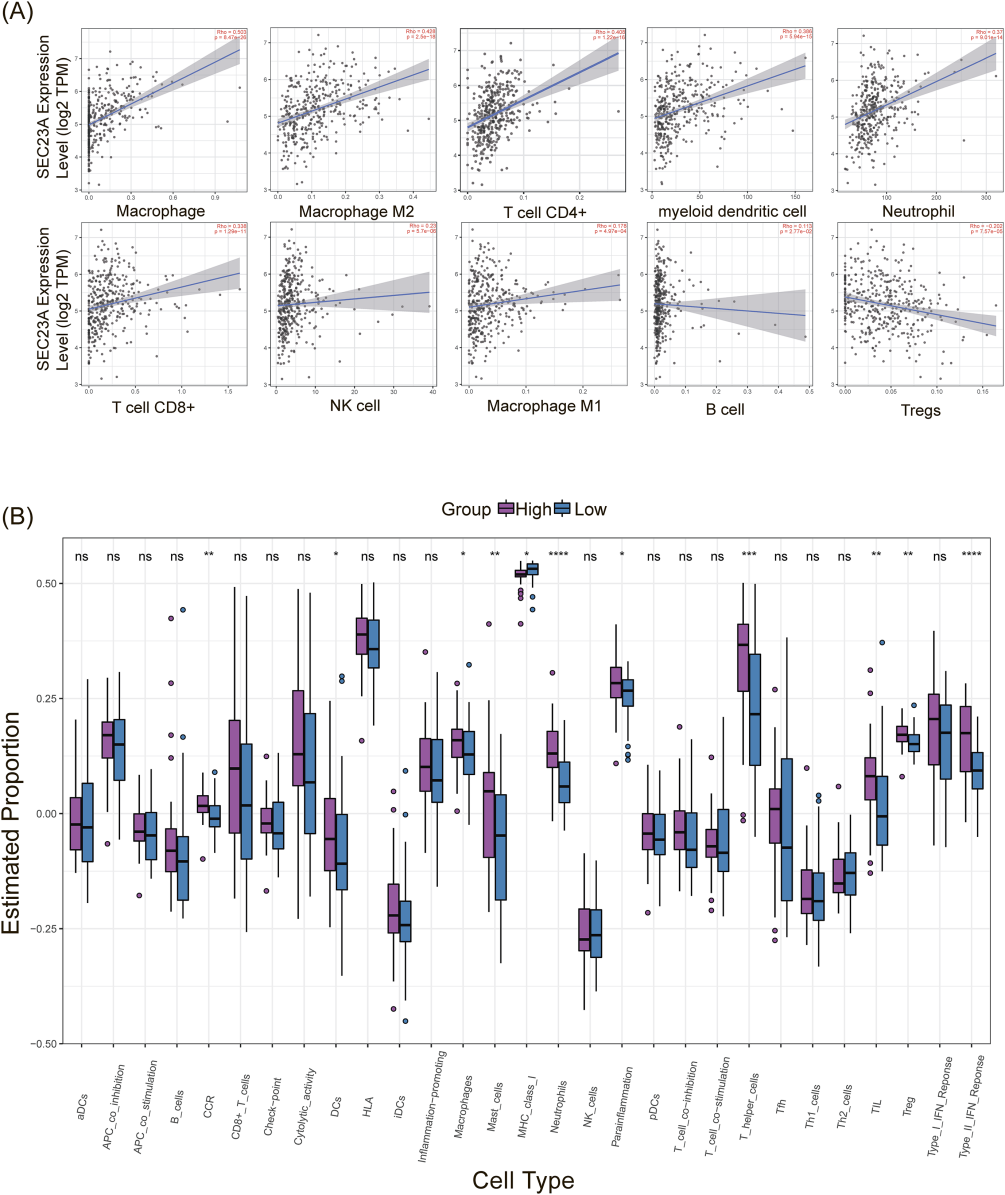
Immunological significance based on Timer 2.0 and ssGSEA. **(A)** Association between immune infiltrates and SEC23A gene expression. **(B)** Comparison of enrichment scores of immune cells and immune-related pathways.

Based on the analysis of the TIMER2.0 database, the relationship between SEC23A and immune cell infiltration in gastric cancer was explored. The results showed positive correlations between SEC23A expression and the infiltration of various immune cells, including macrophages (r = 0.503, *P* = 8.47e-26), M2 macrophages (r = 0.428, *P* = 2.5e-18), CD4^+^ T cells (r = 0.408, *P* = 1.22e-16), myeloid dendritic cells (r = 0.386, *P* = 6.96e-15), neutrophils (r = 0.37, *P* = 9.01e-14), CD8^+^ T cells (r = 0.338, *P* = 1.29e-11), NK cells (r = 0.23, *P* = 5.7e-06), M1 macrophages (r = 0.178, *P* = 4.97e-04), and B cells (r = 0.113, *P* = 2.77e-02). Notably, SEC23A expression was negatively correlated with the infiltration of Treg cells (r = -0.202, *P* = 7.57e-05) (**Figure 5A**).

**Figure 6:**
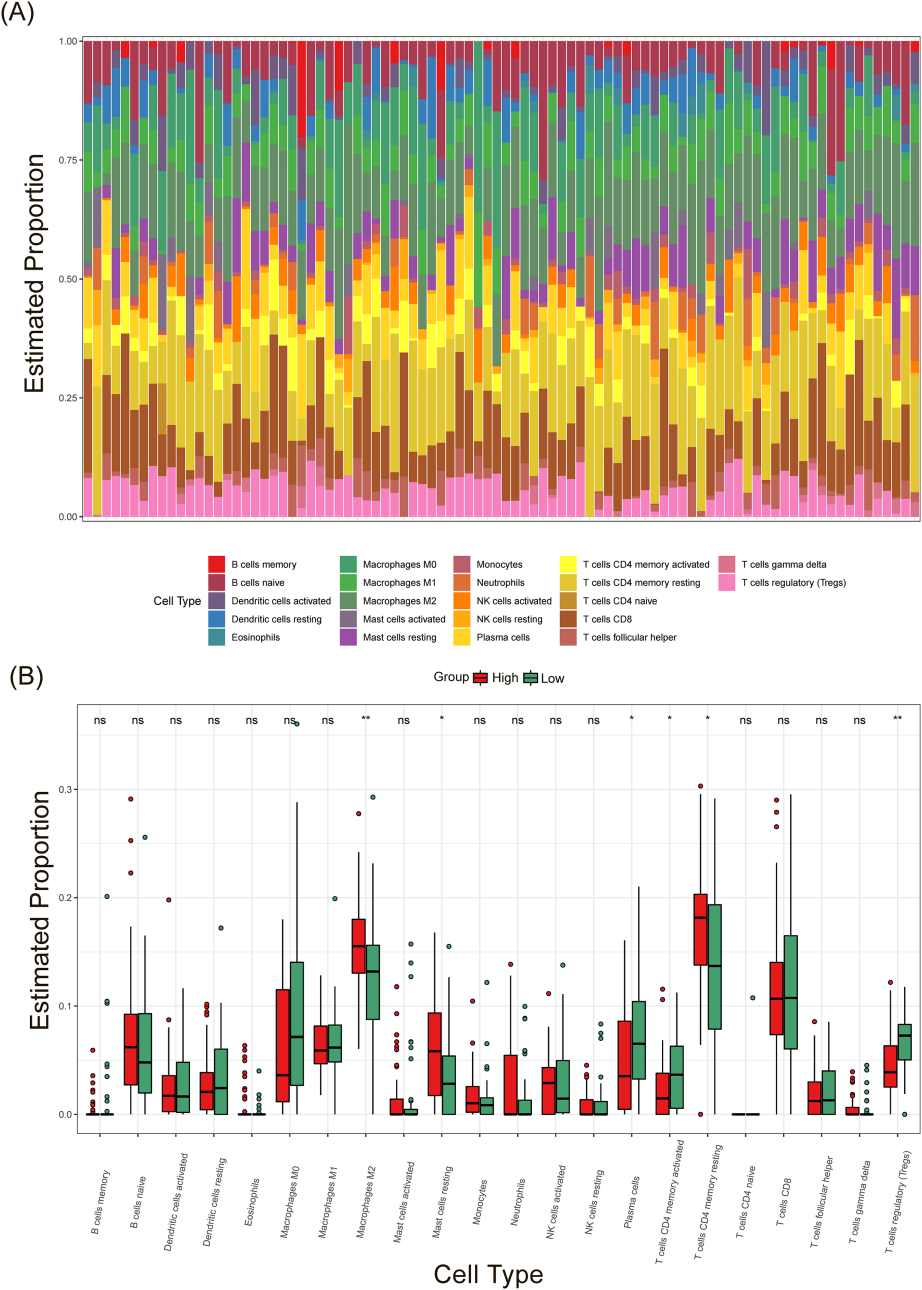
Immunological significance based on CIBERSORT. **(A)** Heatmap and **(B)** Bar plot of immune cell subtype abundance.

Furthermore, immune enrichment analysis using single-sample gene set enrichment analysis (ssGSEA) revealed higher infiltration of various immune cells, including dendritic cells (DCs), macrophages, mast cells, neutrophils, T helper cells, tumor-infiltrating lymphocytes (TIL), and Treg cells, in the SEC23A-highexpression group compared to the SEC23A-low expression group (**Figure 5B**). Additionally, the CIBERSORT algorithm was utilized to further analyze the subtypes of immune cells. The results confirmed that the M2 phenotype of macrophages was more abundant in the SEC23A-high subgroup, while Treg cells were less abundant (**Figures 6A, B**), which aligns with the TIMER2.0 results.

Moreover, immune-related pathways such as chemokine receptors (CCR), para-inflammation, and Type II interferon (IFN) response were found to be significantly upregulated in the SEC23A-high subgroup (**Figure 5B**), indicating potential immunological significance associated with SEC23A expression in GC.

### 3.6 Protein-protein interaction analysis

**Figure 7:**
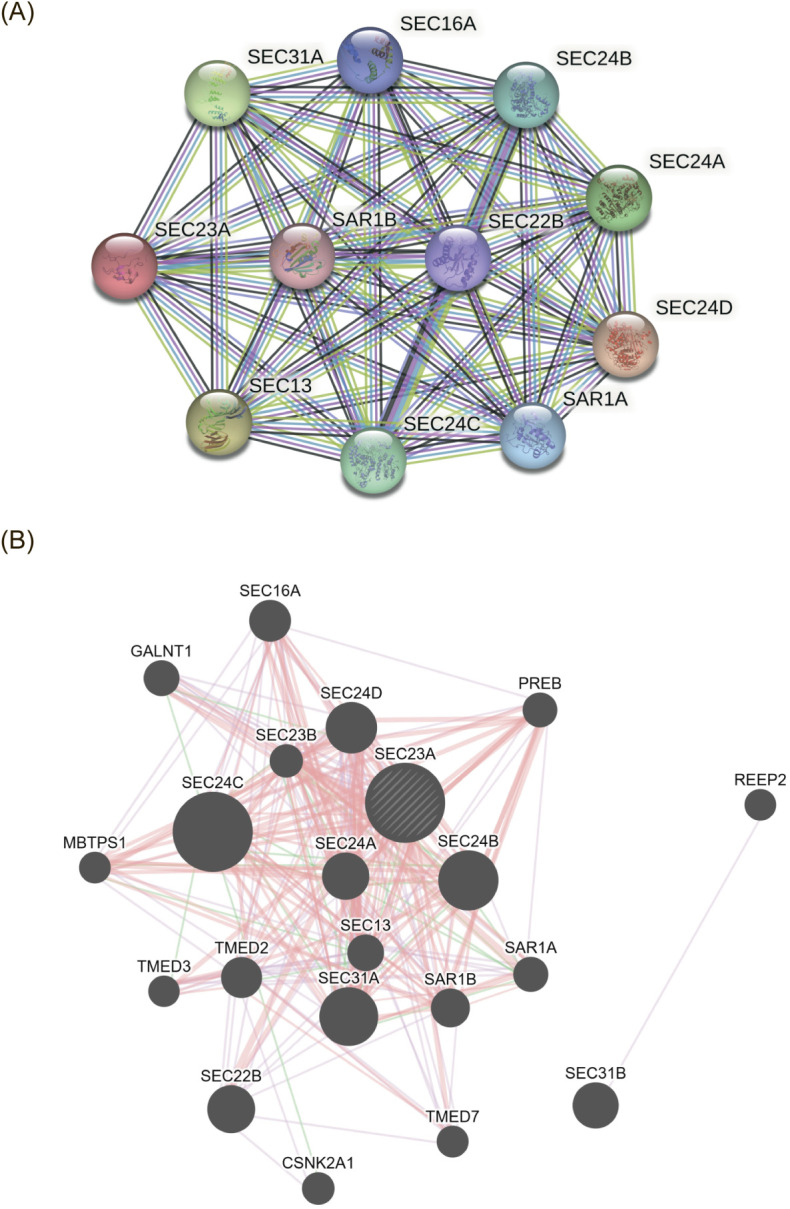
Protein-protein interaction analysis. **(A)** Interaction network of SEC23A with 10 proteins. **(B)** Interaction analysis of SEC23A using GeneMANIA.

To further investigate the possible mechanisms of SEC23A in gastric cancer (GC), an analysis of interacting proteins was conducted. Using the STRING online tool, 10 proteins that were found to interact with SEC23A were identified. These interactions were supported by either experimental evidence or predictions (**Figure 7A**). Additionally, the GeneMANIA database was utilized to perform an interaction analysis on SEC23A. The results obtained were consistent with those from the STRING analysis, indicating that SEC23A mainly interacts with the SEC23/24 family of proteins. This finding suggests that the interaction with homologous proteins within the same family may serve as the foundation for SEC23A to exert its function in GC (**Figure 7B**). Further analysis and exploration of these interactions may contribute to a better understanding of the molecular mechanisms involving SEC23A in GC.

### 3.7 Regulatory mechanism of SEC23A in gastric cancer

**Figure 8:**
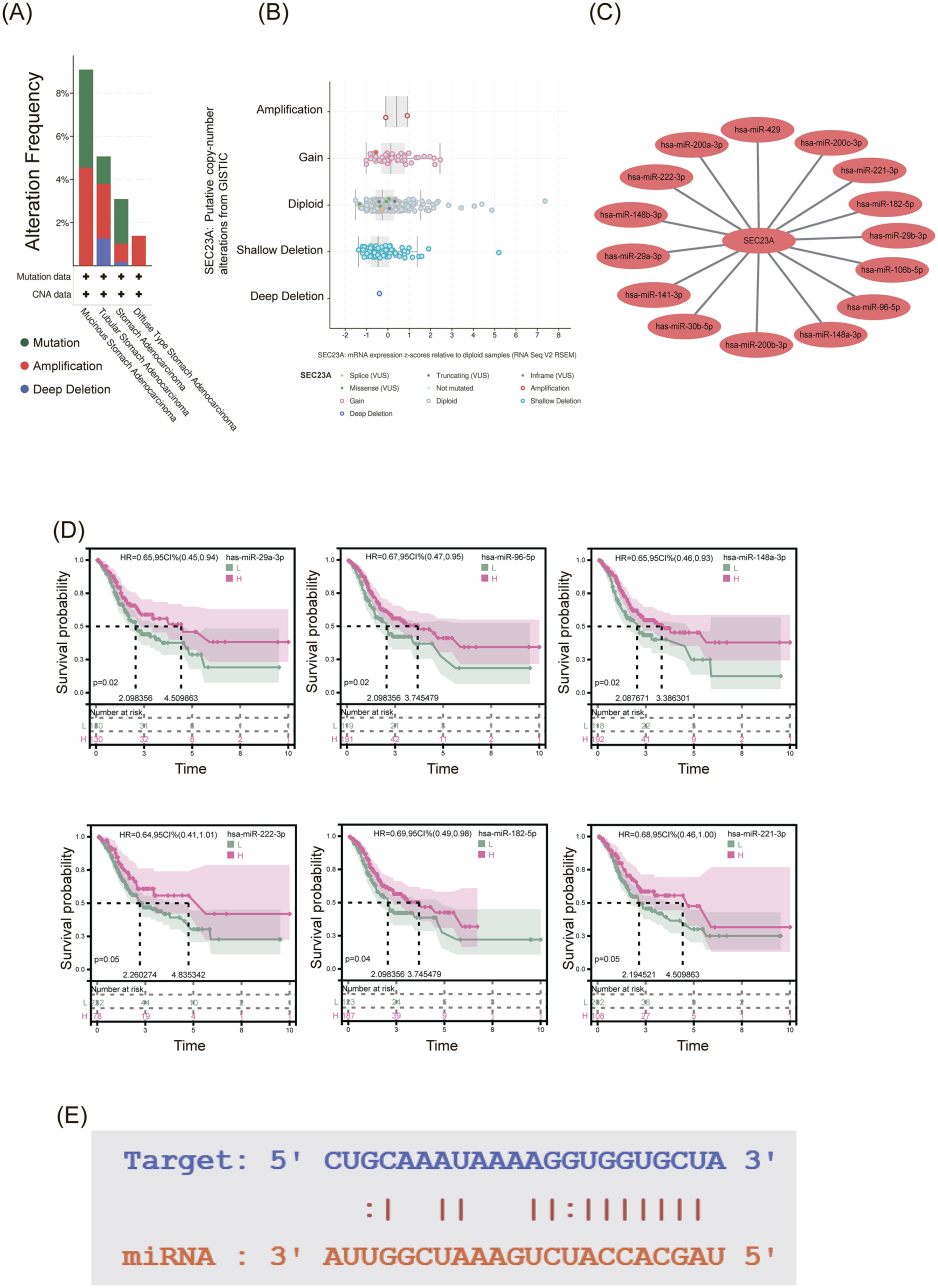
Regulatory mechanism of SEC23A in gastric cancer. **(A, B)** Overall low frequency of the SEC23A genomic mutation. **(C)** ceRNA network predicted 15 miRNAs downregulating SEC23A expression (P < 1.0e-6). **(D)** Correlation between miRNA and prognosis in gastric cancer. **(E)** Binding site of miR-29a-3p on SEC23A.

To investigate the regulatory mechanism of SEC23A overexpression in GC, genetic alterations of SEC23A were analyzed. The results showed that the overall mutation rate of SEC23A in gastric cancer (n = 777) was 3% (**Supplementary Figure 1**). Furthermore, the types of SEC23A gene alterations varied across different types of GC (**Figure 8A**). Copy number variation analysis indicated that gain and amplification had a slight effect on SEC23A expression (**Figure 8B**). However, the overall low frequency of gene alterations cannot explain the high expression of SEC23A in gastric cancer. Therefore, we constructed a ceRNA network. Computational experiments and results have demonstrated that the ceRNA networks can capture important regulatory mechanism of cancer and provide new insights into cancer treatment (18). Therefore, the ceRNA network was constructed based on the miRNA and SEC23A expression matrix from the STRABASE database to predict the miRNAs that could regulate SEC23A mRNA. The network is shown in **Figure 8C**. The ceRNA network predicted 15 miRNAs that downregulate the expression of SEC23A with a statistical significance of *P* < 1.0e-6 (**Table 2**). Lastly, the correlation between miRNA expression and prognosis was analyzed. The results showed that low expression of six miRNAs was associated with poor prognosis (**Figure 8D**). Among these miRNAs, miR-29a-3p exhibited the best prognosis, and its interaction sites with SEC23A are shown in the **Figure 8E**.

**Table 2:** ceRNA network of SEC23A.

Gene	miRna	r	p
SEC23A	hsa-miR-141-3p	-0.47	8.43E-22
SEC23A	hsa-miR-96-5p	-0.458	1.14E-20
SEC23A	hsa-miR-200c-3p	-0.455	2.02E-20
SEC23A	hsa-miR-200b-3p	-0.429	4.62E-18
SEC23A	hsa-miR-429	-0.427	5.89E-18
SEC23A	hsa-miR-200a-3p	-0.417	4.11E-17
SEC23A	hsa-miR-222-3p	-0.396	1.93E-15
SEC23A	hsa-miR-148a-3p	-0.38	3.29E-14
SEC23A	hsa-miR-106b-5p	-0.377	5.48E-14
SEC23A	hsa-miR-182-5p	-0.355	1.66E-12
SEC23A	hsa-miR-221-3p	-0.353	2.49E-12
SEC23A	hsa-miR-29a-3p	-0.347	6.08E-12
SEC23A	hsa-miR-30b-5p	-0.34	1.70E-11
SEC23A	hsa-miR-148b-3p	-0.34	1.64E-11
SEC23A	hsa-miR-29b-3p	-0.334	3.88E-11

### 3.8 External data and experimental validation

**Figure 9:**
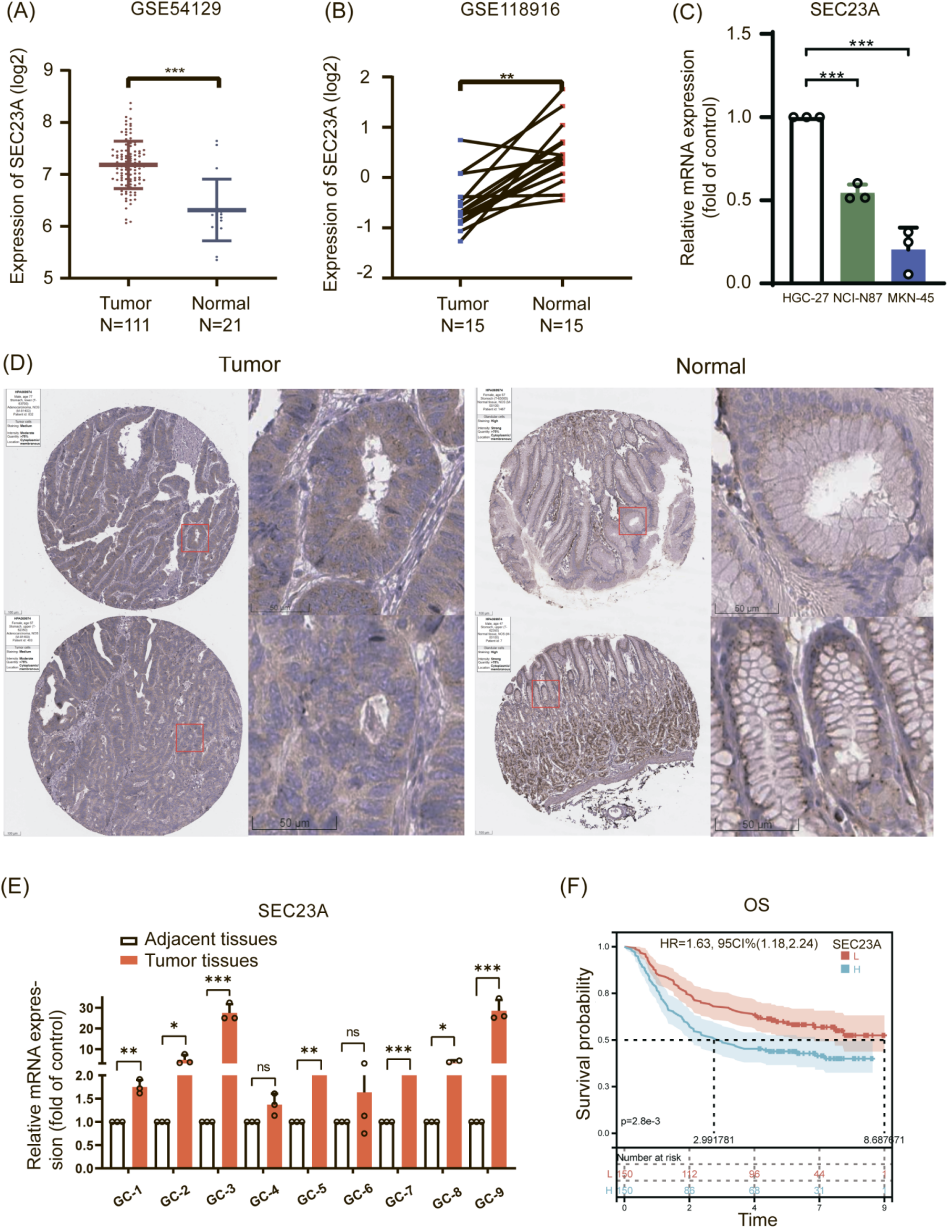
External data and experimental validation. **(A, B)** SEC23A mRNA levels in GC tissues and normal tissues in the GSE54129 and GSE118916 datasets. **(C)** Expression levels of SEC23A in HGC-27, NCI-N87, and MKN-45 cells. **(D)** Images of SEC23A expression in normal and cancerous gastric tissues obtained from The Human Protein Atlas. **(E)** SEC23A mRNA expression measured by RT-qPCR in gastric cancer. **(F)** Survival analysis of SEC23A in the GEO dataset. ***P < 0.001, **P < 0.01, *P < 0.05, ns: not significant.

To validate the aforementioned analysis results, two datasets (GSE54129 and GSE118916) from the GEO database were selected. These datasets included samples of gastric cancer (GC) tissue and normal gastric tissue. The results of the analysis confirmed a significant increase in SEC23A mRNA expression levels in GC tissue compared to normal gastric tissue, which was found to be negatively correlated with patient survival (**Figures 9A, B, F**). Furthermore, the findings revealed that undifferentiated gastric cancer cell lines (HGC-27) exhibited higher expression of SEC23A compared to poorly differentiated gastric cancer cell lines (NCI-N87, MKN-45), thereby suggesting a positive correlation between SEC23A expression and the malignancy of gastric cancer (**Figure 9C**). Moreover, immunohistochemical analysis between GC and normal gastric tissue, based on the HPA database, confirmed the high expression of the protein encoded by SEC23A in gastric cancer tissue (**Figure 9D**). Additionally, the results from the immunofluorescence assay indicated that SEC23A predominantly localizes in the cytoplasm of U-2 cells (**Supplementary Figure 2**). Further RT-qPCR experiments provided additional confirmation, demonstrating a significantly higher expression level of SEC23A mRNA in GC compared to normal gastric tissue (**Figure 9E**). These findings were consistent with the analyses of the GEO data and the TCGA database.

### 3.9 Potential drug prediction

**Figure 10:**
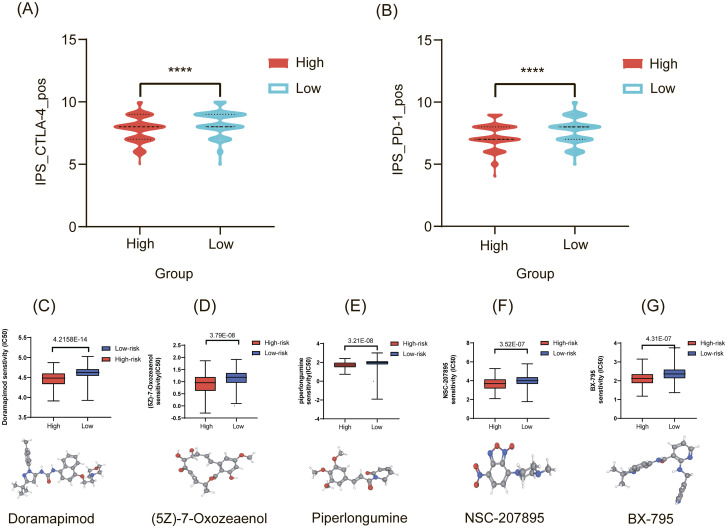
Drug sensitivity analysis. **(A, B)** Reduced sensitivity to both anti-PD1 and anti-CTLA-4 treatments in high-SEC23A groups. **(C–G)** Five small-molecule drugs significantly correlated with SEC23A expression levels. The corresponding 3D structures of these drugs are provided for visual reference.

Survival rates of tumor patients have been markedly improved through immune checkpoint blockade therapy. The findings revealed that the cohort with high SEC23A expression exhibited reduced sensitivity to both anti-PD1 and anti-CTLA-4 treatments (**Figures 10A, B**). These results indicate that the high-expression group of SEC23A may display diminished responsiveness to immunotherapy drugs, potentially attributable to increased infiltration of M2 macrophages and decreased infiltration of Treg cells. Furthermore, a small-molecule drug library was utilized to predict SEC23A-sensitive drugs, revealing significant correlations between drug efficacy and SEC23A expression levels. Notably, compounds such as Doramapimod, a p38/MAPK signaling inhibitor; (5Z)-7-Oxozeaenol, a TAK1 and VEGF-R2 inhibitor; Piperlongumine, an ERK1/2 pathway inhibitor; NSC-207895, a p53 activator; and BX-795, a PDK1 inhibitor, demonstrated pronounced associations with SEC23A expression levels. (**Figures 10C–G**). These findings suggest that these small-molecule targeted drugs represent promising candidates that warrant further investigation for treating patients with high SEC23A expression. Future preclinical studies and clinical trials are necessary to validate their efficacy and safety in gastric cancer.

## 4 Discussion

The SEC23A protein is known to be involved in the transport of secretory proteins within the endoplasmic reticulum and the Golgi apparatus, which has an impact on various cellular functions (7). Recent studies have suggested that SEC23A-transported S100A8 protein can inhibit metastatic colonization through autophagy (8). Furthermore, SEC23A is found to participate in chondrogenesis by regulating the secretome (19, 20). Studies have also indicated that SEC23A can inhibit melanoma metastasis by regulating the tumor microenvironment (TME) (11). Additionally, SEC23A has been implicated in bladder cancer development through the MAPK signaling pathway (21). However, the precise role of SEC23A expression in the mechanism of gastric carcinogenesis remains unclear. Therefore, the aim of our study is to investigate the expression, prognosis, immunological significance, function, and mechanism of SEC23A in GC development. By examining these factors, we hope to gain a better understanding of the potential role of SEC23A in this disease and to identify potential targets for the development of new therapies to treat GC.

Multiple databases, including GEPIA2, GEO, and Kaplan-Meier plotter, in addition to other research studies, were utilized to evaluate SEC23A expression and its clinical prognosis in various tumors. TCGA analysis revealed that SEC23A was highly expressed in GC. Additionally, analysis of GEO and HPA databases, along with RT-qPCR, confirmed a significant increase in SEC23A mRNA expression in GC compared to adjacent tissues. Similar findings have been reported in other studies (22).

To further investigate the clinical significance of SEC23A in GC, its correlation with overall survival (OS), post-progression survival (PPS), and first progression (FP) of GC patients was analyzed using GEPIA2 and Kaplan-Meier plotter. The results revealed that high expression of SEC23A was associated with poor prognosis and shorter overall survival in GC. Specifically, in patients with HER2-negative, T3 stage, and N (1 + 2 + 3) stage gastric cancer, increased SEC23A expression was significantly correlated with poor prognosis. This suggests that SEC23A could potentially serve as a prognostic biomarker for GC. The altered expression of SEC23A may be involved in the development of gastric cancer and may have implications for patient prognosis. Overall, alterations in SEC23A expression levels are implicated in the pathogenesis and progression of gastric cancer, potentially influencing patient prognosis. These findings offer important clues for subsequent investigations into the role of SEC23A in gastric cancer.

To examine the molecular function and underlying mechanism of SEC23A in gastric cancer development, we conducted GO enrichment analysis and Gene Set Enrichment Analysis (GSEA) to investigate gastric cancer-related pathways. In the GO enrichment analysis of differentially expressed genes (DEGs) between the SEC23A-high and SEC23A-low subgroups, we found that the high SEC23A group exhibited functions primarily related to “nuclear and nucleocytoplasmic transport,” “voltage-gated channel activity,” “transcriptional regulatory complex,” “positive regulation of cytokine production,” and “epithelial tube morphogenesis”among. Unlike previous studies, our focus was more on changes in gastric cancer occurrence and progression, in addition to immune-related pathways, in the GSEA results (22). In the GSEA results, several pathways associated with cell proliferation, differentiation, signal transduction, and immunity were enriched in samples with high expression of SEC23A. These pathways included TGF-β, MAPK, and CCR5 signaling pathways (23, 24). TGF-β in the tumor microenvironment of the stomach can promote the differentiation and expansion of regulatory T cells (Tregs) and M2 macrophages (25, 26). The MAPK signaling pathway, activated by silk gland factor activation protein kinases, is an important information transmission chain within cells. It involves Ser/Thr protein kinases that are widely expressed and participate in various cellular physiological activities, such as growth, development, differentiation, and apoptosis. This pathway is a major hotspot for inducing tumor formation. CCR5 can promote the migration of inflammatory cells, and several chemokines have been shown to have predictive value for the prognosis of gastric cancer patients. Therefore, SEC23A may be involved in regulating these signaling pathways to promote gastric cancer progression and reduce the survival rate of gastric cancer patients.

To delve deeper into the role of SEC23A in gastric cancer cells, the expression levels of SEC23A in the AGS and HGC-27 gastric cancer cell lines were suppressed using siRNA. The colony formation assay and the EdU (5-ethynyl-2’-deoxyuridine) assay were utilized to evaluate alterations in cellular proliferation capacity. The colony formation assay demonstrated a notable decrease in the quantity of colonies formed by AGS and HGC-27 cells with suppressed SEC23A expression, highlighting that the downregulation of SEC23A hampers the proliferation capacity of gastric cancer cells. The outcomes of the EdU assay indicated a substantial decline in the percentage of EdU-positive cells in AGS and HGC-27 cells with reduced SEC23A levels compared to the control group, providing additional validation of the inhibitory impact of SEC23A downregulation on the proliferation capacity of gastric cancer cells. These observations suggest that SEC23A could potentially serve as a positive regulator in the proliferation mechanism of gastric cancer cells, with its decreased expression possibly linked to reduced gastric cancer cell proliferation. Overall, these findings suggest that SEC23A may exert a positive regulatory role in the proliferation of gastric cancer cells, with low expression of SEC23A being related to reduced proliferation of gastric cancer cells, a correlation that aligns with the above pathway enrichment results.

The interaction between tumor cells and the tumor microenvironment (TME) plays a critical role in tumor metastasis. It has been shown that immune cell infiltration is key to the development and prognosis of cancer (27, 28). Our analysis using TIMER2.0, ssGSEA, and CIBERSORT showed that SEC23A was primarily positively correlated with the infiltration of M2 macrophages and negatively correlated with Treg cell infiltration. This suggests that SEC23A may play a role in regulating the TME. M2 macrophages and Treg cells are known to mediate an immunosuppressive TME. M2 macrophages can disrupt the matrix membrane of endothelial cells to promote tumor migration. They also secrete the anti-inflammatory cytokine TGF-β, which activates cancer-associated fibroblasts (CAFs) and promotes tumor progression (29, 30). Our findings indicate that SEC23A may regulate immune cell infiltration in the TME through the TGF-β pathway, as we consistently observed upregulation of TGF-β signaling in the SEC23A high-expression group. Consistent with our findings, a recent study investigated vesicle-mediated transport-related genes in gastric cancer and identified SEC23A as the dominant gene in their prognostic model (31). They reported that high SEC23A expression correlated with increased M2 macrophage infiltration and an immunosuppressive microenvironment, providing independent validation of our observations. Additionally, our analysis of immune-related pathways revealed the activation of para-inflammatory and low-grade inflammation in groups with high SEC23A expression, which is associated with cancer progression (32). Studies have shown that SEC23A positively correlates with Immune score and regulates the MAPK signaling pathway, thereby influencing the immune microenvironment in bladder cancer (21). Additionally, SEC23A has been reported to remodel the immune microenvironment by regulating secreted proteins such as S100A8, which inhibits metastatic colonization through autocrine activation of autophagy (8). These findings provide a mechanistic foundation for the association between SEC23A and tumor immunity observed in our study.

We also explained the possible mechanism of SEC23A from the perspective of protein-protein interactions. Our findings revealed a predilection for SEC23A to predominantly engage in binding interactions with homologous family proteins. This preference substantiates the notion that SEC23A fulfills its main functions by forging associations with proteins within its homologous family.

Through the modulation of gene expression, miRNAs have been implicated in the pathogenesis of gastric cancer (33). In order to elucidate the underlying regulatory mechanism of SEC23A in gastric cancer development, we constructed a ceRNA network. Within this network, we identified 15 miRNAs that exhibit a negative regulatory effect on the expression of SEC23A. Subsequently, we conducted a comprehensive analysis of the correlation between these miRNAs and the prognosis of gastric cancer. Remarkably, our findings revealed that six distinct miRNA types are significantly associated with an unfavorable prognosis in gastric cancer cases. Current studies have found that miR-29a-3p-dependent COL3A1 and COL5A1 expression reduction assists sulforaphane to inhibit gastric cancer progression (34). Additionally, our analysis revealed a notable downregulation of miR-29a-3p expression in gastric cancer tissues when compared to corresponding non-tumor tissues (35). This observation is consistent with the observed upregulation of SEC23A expression in gastric cancer. Furthermore, we discovered that miR-148a-3p acts upon CEMIP to suppress the formation and progression of gastric cancer cells, thus emerging as a promising screening biomarker for gastric cancer (36, 37). Moreover, the downregulation of miR-182-5p has also been noted in gastric cancer (38). Consequently, it is plausible that these aforementioned miRNAs exert a negative regulatory influence on the expression levels of SEC23A, consequently influencing the overall prognosis of gastric cancer.

In recent years, the efficacy of immunotherapy and molecular targeted therapy in gastric cancer has been remarkable (39, 40). In this study, we utilized predictive analysis to evaluate the efficacy of immune checkpoint drugs and chemotherapeutic agents. Our results demonstrated that patients with high expression levels of SEC23A exhibited lower sensitivity to anti-PD-1 therapy and anti-CTLA-4 therapy. As a result, immunotherapy utilizing monoclonal antibodies targeting PD-1 and CTLA-4 may be less effective for gastric cancer patients with elevated SEC23A expression. Regarding therapeutic response, a study identified SEC23A as one of eight prognostic mitochondrial autophagy-related genes in gastric cancer using LASSO-Cox regression analysis (41). Their risk score was associated with immune cell infiltration and showed potential value in guiding personalized immunotherapy. Furthermore, we identified small-molecule inhibitors with greater sensitivity toward the high-expression group of SEC23A through our drug prediction analyses. Notably, NSC-207895 has been demonstrated to enhance the anti-tumor activity mediated by the p53 protein (42). Moreover, BIRB-0796 acts as a potent inhibitor of p38 MAPK, and subsequent gene set enrichment analyses revealed a strong association between SEC23A and the MAPK pathway. Importantly, our predictive analyses indicate that the high-expression group of SEC23A may exhibit heightened sensitivity to a variety of anti-tumor drugs, thereby providing novel insights into potential neoadjuvant and immunotherapeutic approaches for gastric cancer. However, as these predictions are based on computational algorithms, they require rigorous experimental validation in preclinical models and, ultimately, clinical trials.

While our study provides a comprehensive bioinformatic analysis and preliminary *in vitro* validation of SEC23A’s role in gastric cancer, several limitations must be acknowledged. First, the mechanistic conclusions, particularly those linking SEC23A to specific signaling pathways (e.g., TGF-β, MAPK) and immune cell modulation (e.g., M2 macrophage polarization), are largely derived from correlative analyses and pathway enrichment. They remain hypothesis-generating and require rigorous experimental validation. Functional studies, such as co-culture systems to directly assess the impact of SEC23A manipulation on macrophage polarization or T cell activity, and *in vivo* animal models to evaluate its effect on tumor growth and the immune microenvironment, are essential next steps. Based on our current findings, we hypothesize that SEC23A may regulate the secretion of TGF-β or other cytokines from gastric cancer cells, thereby promoting M2 macrophage polarization. This hypothesis will be tested in future work using Transwell co-culture assays, TGF-β neutralizing antibodies, and macrophage marker analysis. Second, our *in vitro* experiments were limited to proliferation assays in two cell lines. The functional role of SEC23A in other cancer hallmarks, such as metastasis, invasion, and therapy resistance, remains to be explored. Third, the small sample size of our clinical validation cohort (n = 9) and the inherent heterogeneity of public databases mean that our findings should be interpreted with caution and validated in larger, independent, and diverse patient cohorts. Finally, the predicted drug sensitivities, while intriguing, are based on computational algorithms and require preclinical testing in relevant models before any clinical translation can be considered. Future work should focus on addressing these limitations to firmly establish SEC23A as a viable therapeutic target.

## 5 Conclusion

The bioinformatics analysis revealed the potential mechanism of SEC23A overexpression and its role in promoting gastric cancer development by modulating tumor immunity. Experimental validation confirmed that SEC23A promotes the proliferation of gastric cancer cells both *in vitro* and *in vivo*. Drug sensitivity analysis indicated that gastric cancer patients with SEC23A overexpression exhibit lower sensitivity to PD-1 and CTLA-4 monoclonal antibodies but higher sensitivity to specific small-molecule inhibitors. These findings suggest that SEC23A could potentially serve as a novel target for gastric cancer therapy.

## Data Availability

The original contributions presented in the study are included in the article/**Supplementary Material**. Further inquiries can be directed to the corresponding authors.

## Supplementary material

Supplementary Figure 1 Genetic alterations of SEC23A. **(A)** The overall mutation rate of SEC23A in gastric cancer. **(B)** Mutations in distinct domains of SEC23A.

Supplementary Figure 2 Subcellular studies on SEC23A. The target protein of the SEC23A gene was mainly located in the nucleoplasm and vesicles of the U-2 tumor cell line.
