# Supplementary material for: The vesicle transport gene SEC23A is a novel prognostic indicator and therapeutic target in gastric cancer
Source: Front Oncol. 2026 Apr 13;16:1728472. doi: 10.3389/fonc.2026.1728472 (PMC13111058; doi:10.3389/fonc.2026.1728472)
Supplement: Supplementary Figure 1 — Genetic alterations of SEC23A. (A) The overall mutation rate of SEC23A in gastric cancer. (B) Mutations in distinct domains of SEC23A. [file DataSheet1.pdf]

(A)

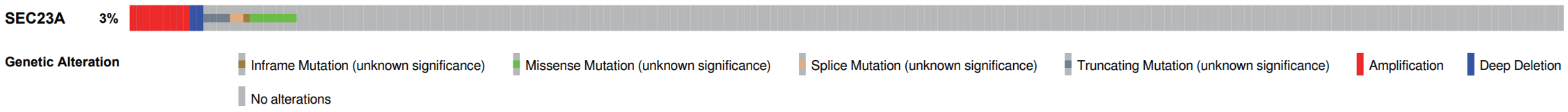

(B)

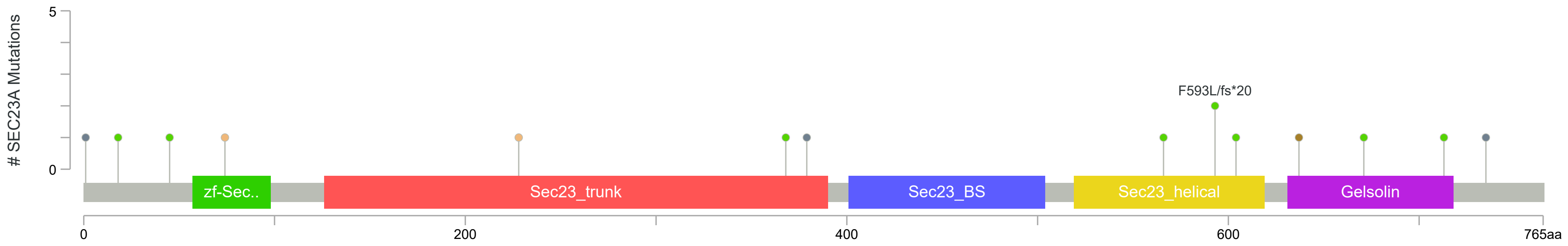

Supplementary Figure 1 (A)The overall mutation rate of SEC23A in gastric cancer. (B)Mutations in distinct domains of SEC23A.

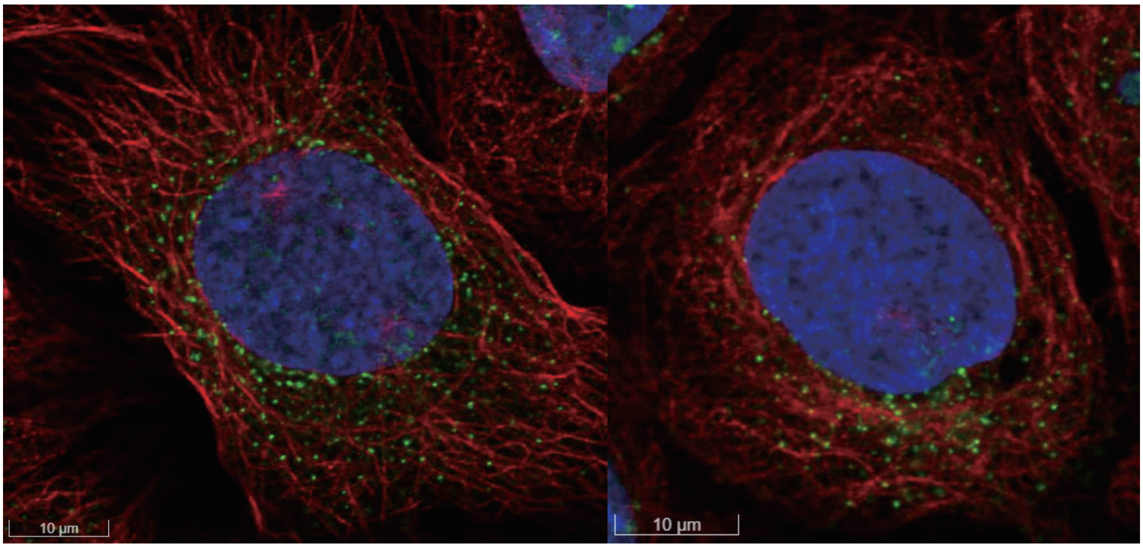

Supplementary Figure 2 Subcellular studies on SEC23A. The target protein of the SEC23A gene was mainly located in the Nucleoplasm and Vesicles of the U-2 tumour cell line.
